# A Single Tim Translocase in the Mitosomes of *Giardia intestinalis* Illustrates Convergence of Protein Import Machines in Anaerobic Eukaryotes

**DOI:** 10.1093/gbe/evy215

**Published:** 2018-09-28

**Authors:** Eva Pyrihová, Alžběta Motyčková, Luboš Voleman, Natalia Wandyszewska, Radovan Fišer, Gabriela Seydlová, Andrew Roger, Martin Kolísko, Pavel Doležal

**Affiliations:** 1 Department of Parasitology, Faculty of Science, Charles University, Vestec, Czech Republic; 2 Department of Genetics and Microbiology, Charles University, Praha 2, Czech Republic; 3 Centre for Comparative Genomics and Evolutionary Bioinformatics, Department of Biochemistry and Molecular Biology, Dalhousie University, Halifax, Canada; 4 Biology Centre CAS, České Budějovice, Czech Republic

**Keywords:** mitochondrial evolution, protein transport, TIM translocase, Tim17, mitosomes, Giardia, anaerobic protists

## Abstract

Mitochondria have evolved diverse forms across eukaryotic diversity in adaptation to anoxia. Mitosomes are the simplest and the least well-studied type of anaerobic mitochondria. Transport of proteins via TIM complexes, composed of three proteins of the Tim17 protein family (Tim17/22/23), is one of the key unifying aspects of mitochondria and mitochondria-derived organelles. However, multiple experimental and bioinformatic attempts have so far failed to identify the nature of TIM in mitosomes of the anaerobic metamonad protist, *Giardia intestinalis*, one of the few experimental models for mitosome biology. Here, we present the identification of a single *G. intestinalis* Tim17 protein (GiTim17), made possible only by the implementation of a metamonad-specific hidden Markov model. While very divergent in primary sequence and in predicted membrane topology, experimental data suggest that GiTim17 is an inner membrane mitosomal protein, forming a disulphide-linked dimer. We suggest that the peculiar GiTim17 sequence reflects adaptation to the unusual, detergent resistant, inner mitosomal membrane. Specific pull-down experiments indicate interaction of GiTim17 with mitosomal Tim44, the tethering component of the import motor complex. Analysis of TIM complexes across eukaryote diversity suggests that a “single Tim” translocase is a convergent adaptation of mitosomes in anaerobic protists, with Tim22 and Tim17 (but not Tim23), providing the protein backbone.

## Introduction

The endosymbiotic acquisition of mitochondria (Roger et al. 2017) was a key event in the evolution of eukaryotes. The establishment of an effective system for protein import from the cytosol into mitochondria involved both, the adaptation of the original endosymbiont translocases and the creation of eukaryote-specific protein transport complexes (Dolezal et al. 2006; Fukasawa et al. 2017; Vitali et al. 2018). In canonical mitochondria, the protein import machinery is a complex network of specialized protein translocases, comprising >35 different protein components (Dudek et al. 2013).

The unicellular anaerobic parasite, *G.intestinalis*, possesses highly reduced mitochondria, tiny organelles called mitosomes. Currently, their only known function is iron–sulfur cluster synthesis through the ISC pathway (Tovar et al. 2003). Mitosomes have lost most other canonical mitochondrial functions (Jedelský et al. 2011). They lack a genome and are devoid of cristae; yet, they are still surrounded by two membranes (Tovar et al. 2003).

Canonical mitochondria employ several independent types of protein transport systems, including the TOM and SAM complexes in the outer membrane, the MIA pathway in the intermembrane space, and the TIM23 and TIM22 complexes transporting proteins across or into the inner membrane, respectively (Dudek et al. 2013). Proteins from the Tim17/22/23 protein family form the core of both TIM complexes. The protein-conducting channel of the TIM23 complex is formed by two Tim17/22/23 family proteins, Tim23 and Tim17 (Mokranjac and Neupert 2010). Transport through the TIM23 complex is initially energized by membrane potential, whereas translocation is driven by the mtHsp70 chaperone (Chacinska et al. 2009). Mitochondrial Hsp70 is part of the PAM motor complex, which is tethered to the TIM23 complex via the Tim44 protein (Schneider et al. 1994). The channel of the TIM22 complex is formed by a single Tim17 family protein, Tim22, and the TIM22 translocase requires only energy from the membrane potential to insert proteins into the inner mitochondrial membrane (Kovermann et al. 2002).

The presence of similar protein targeting signals and homologous SAM, TOM, and TIM machineries have been considered crucial supporting evidence for a common origin of mitochondria, mitosomes, and hydrogen-producing hydrogenosomes (Dolezal et al. 2005; Lithgow and Schneider 2010; Shiflett and Johnson 2010; Garg et al. 2015). However, of the three molecular machines, only a minimal TOM complex is known from *Giardia* (Dagley et al. 2009), even though its genome has been fully sequenced (Morrison et al. 2007) and proteomic data from mitosomes are available (Jedelský et al. 2011; Martincová et al. 2015; Rout et al. 2016). Only four components of the import motor complex, PAM, are known. A hidden Markov model (HMM) search identified mitosomal Pam18 (Dolezal et al. 2005), while proteomics of density gradient-derived cell fractions resulted in the identification of Pam16 (Jedelský et al. 2011). These J- and J-like proteins, respectively, modulate the activity of the actual motor molecule mtHsp70 (Dolezal et al. 2005).

Recently, another core component of the mitosomal protein transport, Tim44, was identified using high-affinity coprecipitation of in vivo biotin-tagged mitosomal bait proteins (Martincová et al. 2015).

Despite all of these efforts, the essential channel-forming Tim17 family protein remained elusive in mitosomes. Two alternate hypotheses explaining the absence of a Tim17 family protein in *Giardia* have been drawn: 1) import into mitosomes is facilitated through a lineage-specific protein channel or some other molecular mechanism—this would be in line with the presence of many unique *Giardia*-specific proteins with no clear orthologues in other eukaryotes (Martincová et al. 2015; Rout et al. 2016); or 2) the primary sequence of Tim17 has diverged to the extent that bioinformatic approaches cannot detect any similarity to canonical Tim17 homologs. Given that *Giardia* protein sequences are frequently highly divergent, it is not surprising that bioinformatics approaches often fail to identify clear homology to known mitochondrial components, even when they are present (Collins et al. 2003), as was the case for mitosomal Tom40 (Dagley et al. 2009) and Tim44 (Martincová et al. 2015). The mechanism of protein translocation across the inner mitosomal membrane thus remains one of the “last great mysteries” of these organelles.

Here, we present evidence for the latter hypothesis. By a tailored HMM-based bioinformatic analysis we identified the long sought-after Tim17 orthologue in *Giardia*. Our experiments suggest that this extremely divergent Tim17 functions in the inner mitosomal membrane, where it interacts with other mitosomal protein import components.

## Results and Discussion

**Fig. 1.**
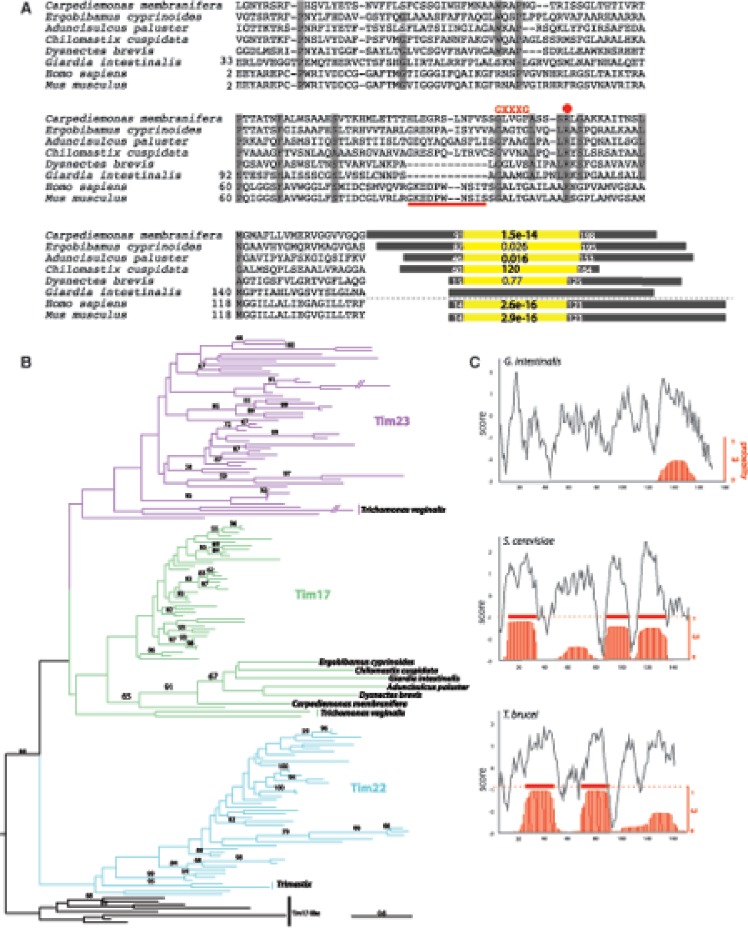
—*Giardia* has a single Tim17 family protein. (*A*) Protein sequence alignment of GiTim17 with the orthologues from other metamonads, *Homo sapiens* and *Mus musculus.* Because of the incomplete N-terminal sequences of metamonads, truncated proteins are shown (positions corresponding to the complete sequences of *G. intestinalis*, *H. sapiens*, and *M. musculus* are shown). Red dot depicts the conserved arginine residue essential for the interaction with Tim44; red line represents the deletion conserved in *G. intestinalis* and *D. brevis*. Diagrams next to the alignment correspond to the particular Tim17 proteins (gray rectangle) with highlighted Tim17/22/23 domain identified by HHpred (Hildebrand et al. 2009) against Pfam (yellow rectangle). The *e*-value and start and end positions of the domain are shown. (*B*) Phylogenetic reconstruction of Tim17, Tim22, and Tim23 proteins including the metamonad sequences. (*C*) Hydrophobicity profiles (grey line) by Protscale (Gasteiger et al. 2005)—(Kyte and Doolittle scale) and transmembrane domain prediction (red lines) by TMHMM (Krogh et al. 2001) of Tim17 proteins from *G. intestinalis*, *Saccharomyces cerevisiae*, and *T. brucei*.

We performed several rounds of hmmsearch against a Metamonada protein database enriched with recently published transcriptomes of *Carpediemonas-*like organisms (CLOs) (Leger et al. 2017) and the predicted proteome of *Giardia* (Aurrecoechea et al. 2017). The initial HMM model was built from a Pfam seed alignment for the Tim17 family (PF02466) and enriched for newly identified sequences after each of the iterations. After the third round, there were no new sequences recovered. This search returned a single *Giardia* Tim17 candidate sequence, GL50803_10452, encoding a protein of 180 amino acids and a predicted molecular mass of 19.4 kDa. Hereafter this protein is referred to as GiTim17. The primary sequence of GiTim17 is extremely divergent relative to homologs, to the extent that even one of the most sensitive protein homology detection tools, HHpred (Alva et al. 2016), failed to recognize this protein as a member of the Tim17/22/23 protein family, whereas all other metamonad sequences were clearly identified as Tim17/22/23 proteins (fig. 1*A*). Enriching the HMM profile with phylogenetically related orthologues was crucial for identification of the GiTim17 candidate (Likic et al. 2010).

Attempts to recover a well-resolved phylogenetic tree of polytopic membranes such as Tim17/22/23 family proteins are hindered by the extreme divergence of the proteins across species (Sojo et al. 2016). In case of Tim17/22/23, the relatively short length of the amino acid sequence also plays a role. However, our phylogenetic analysis has clearly demonstrated, with high statistical support, that GiTim17 is closely related to Tim17 proteins from *Giardia’s* closest relatives, the CLOs (BP support 91, fig. 1*B*, supplementary fig. 1, Supplementary Material online). Moreover, GiTim17 also shares a short deletion between TMD1 and 2 with its closest free-living relative *Dysnectes brevis* (Leger et al. 2017) (fig. 1*A*). These results strongly suggest that GiTim17 is, from an evolutionary standpoint, the previously unidentified Tim17 orthologue in *Giardia*.

**Fig. 2.**
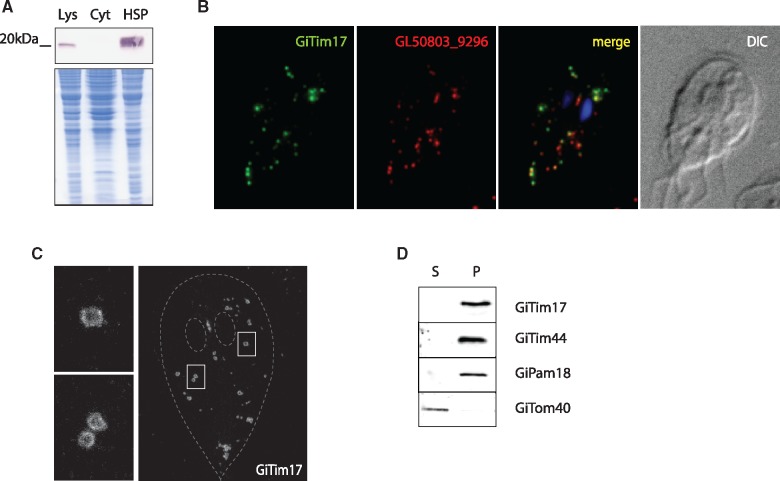
—GiTim17 is an inner mitosomal membrane protein. (*A*) GiTim17 was expressed with a C-terminal HA-tag and the protein was detected by western blot of *G. intestinalis* cellular fractions. The protein was present in the lysate and the high speed pellet fraction, which is enriched for mitosomes. Lys-lysate, Cyt-cytosol, HSP-high speed pellet. (*B*) Mitosomal localization of GiTim17 was confirmed by immunofluorescence microscopy using GL50803_9296 as the mitosomal marker. (*C*) STED microscopy of HA-tagged GiTim17 shows its discrete localization on the periphery of the mitosomes, corresponding to the mitosomal membrane. Two images on the left depict details of the displayed cell. (*D*) Western blot analysis of digitonin-solubilized HSP fraction shows differential distribution of GiTom40 (the outer mitosomal membrane marker) and GiTim17. GiTim17 was found along with GiPam18 and GiTim44, which are associated with the inner mitosomal membrane. S-supernatant, P-pellet.

To test whether GiTim17 is a mitosomal protein, it was expressed with a C-terminal HA-tag in *Giardia*. Western blot analysis showed that GiTim17 is enriched in the high-speed pellet fraction (HSP) containing mitosomes and other membrane-bounded organelles (fig. 2*A*). Moreover, fluorescence microscopy confirmed that GiTim17 colocalizes with mitosomal marker protein, GL50803_9296 (Martincová et al. 2015; fig. 2*B*). Interestingly, GiTim17 could be found among the proteins identified in our earlier proteomic analysis (Martincová et al. 2015); however, it was not recognized at the time as a putative Tim17 homolog. This demonstrates that the endogenous GiTim17 gene is expressed in *Giardia*.

GiTim17 possesses four hydrophobic regions corresponding to the four putative transmembrane domains (TMDs) of canonical Tim17 family proteins (fig. 1*C*) and the overall hydrophobicity corresponds to other Tim17 orthologues (supplementary fig. 2, Supplementary Material online). However, the hydrophobic regions are not recognized as TMDs by widely used HMM-based predictors such as TMHMM [21]. This can likely be attributed to the stringent nature of the diagnostic model in TMHMM predictor. Only one of the four putative TMDs bears the typical glycine zipper (GxxxG) motif for the intramembrane interaction of TMDs (fig. 1*A*). The extreme divergence of putative TMDs in GiTim17 could be explained as a loss of functional membrane insertion or adaptation to different biochemical properties of the mitosomal inner membrane.

The resolution of stimulated emission depletion (STED) microscopy enables discrimination of soluble and membrane-bound proteins in mitochondria (Jakobs and Wurm 2014). Detection of GiTim17 by STED demonstrated its presence specifically on the periphery of mitosomes (fig. 2*C*), thus supporting its insertion into the mitosomal membrane. In order to distinguish whether GiTim17 occupies the outer or inner mitosomal membrane, the organelles were treated with detergent for inner and outer membrane distinction based on their lipid composition. The HSP was incubated in different detergents (digitonin, DDM, deoxycholate, Triton X-114, Zwittergent) and the resulting soluble and insoluble fractions were probed for mitosomal proteins. Repeatedly, the outer mitosomal membrane protein, Tom40, was efficiently solubilized, whereas GiTim17 was always retained in the pellet fraction along with the inner membrane anchored GiPam18 and the peripheral membrane protein GiTim44, as shown for the experiment with 2% digitonin (fig. 2*D*). These results strongly suggest that GiTim17 is indeed localized to the inner mitosomal membrane. However, the overall resistance of the mitosomal inner membrane to detergent treatment suggests that it has a highly unusual lipid composition, as compared with the properties of canonical mitochondria (Schagger and Pfeiffer 2000). In light of these results, it is possible that the nonconformity of putative TMDs in GiTim17 is the result of adaptation to the unusual composition of the mitosomal inner membrane.

**Fig. 3.**
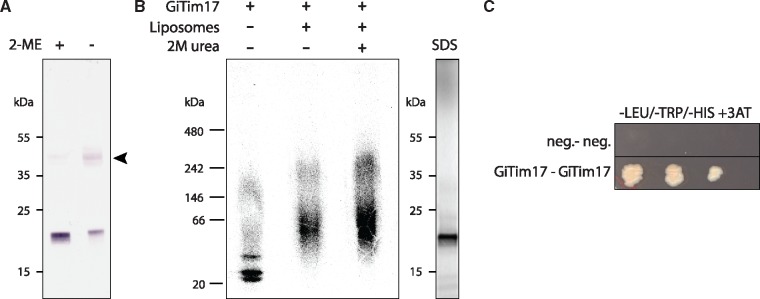
—GiTim17 forms dimers in the mitosomal membrane. (*A*) GiTim17 forms an ∼40 kDa complex on nonreducing SDS-PAGE. The complex depicted by the arrowhead brakes apart in the presence of reducing agent such as 2-mercapthoethanol (2-ME). (*B*) The complex of higher molecular weight corresponding approximately to the dimer of GiTim17 assembled in the liposomes upon in vitro translation. The complex was resistant to 2 M urea, which indicates its membrane insertion. Control SDS-PAGE of translated GiTim17 is shown on the right. (*C*) Mutual interaction of two GiTim17 proteins was positively tested in a yeast two hybrid assay under stringent conditions of 3-amino-1, 2, 4-triazole (3-AT).

Canonical TIM23 complexes comprise of more than one protein from the Tim17/22/23 protein family—Tim17 and Tim23. Furthermore, both TIM22 and TIM23 complexes homodimerize into super assemblies of twin pore architecture (Rehling et al. 2003; Martinez-Caballero et al. 2007). Given that we were able to identify only one member of the family in *Giardia*, we hypothesize that GiTim17 forms dimers in order to form a functional pore. Indeed, three lines of evidence suggest the capability of GiTim17 to dimerize: 1) In vivo, GiTim17 is part of a protein complex, which is bound by a disulphide bond and approximately double the size of a single GiTim17 protein (fig. 3*A*); 2) Upon in vitro translation, it forms a complex of double size in an experimental membrane (fig. 3*B*); and 3) It specifically interacts with itself in a yeast two-hybrid (Y2H) assay (fig. 3*C*).

**Fig. 4.**
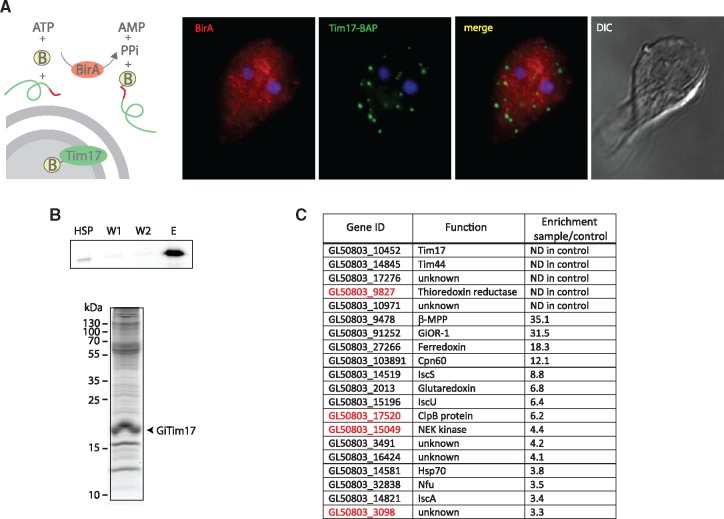
—GiTim17 is localized in proximity to GiTim44. (*A*) BAP-tagged GiTim17 (green) is biotinylated in vivo by the HA-tagged cytosolic BirA (red). (*B*) The proteins chemically cross-linked to GiTim17 by DTME were copurified and analyzed by mass spectrometry. (Top) The detection of biotinylated GiTim17 in the fractions derived from the protein purification. HSP—the initial high-speed pellet fraction, W1 and W2—wash steps, E—eluate from the streptavidin-coated Dynabeads. (Bottom) The SDS-PAGE gel of the elute. (*C*) Identified proteins were ordered according to the enrichment score. Only proteins enriched more than three times are shown (the complete list of proteins is shown in supplementary table 1, Supplementary Material online). Putative new mitosomal proteins are shown in red letters.

The Tim17 family proteins constitute the core of protein-conducting channels, the activity and specificity of which are controlled by other components of the TIM and PAM complexes. Therefore, the interaction of GiTim17 with other mitosomal components was investigated. Unfortunately, without convenient solubilization conditions, the association of GiTim17 within a putative translocation complex could not be tested by blue native PAGE or by coprecipitations under native conditions. Instead, the in vivo biotinylation approach coupled to chemical cross-linking of adjacent sulfhydryl groups by DTME was used to isolate interacting partners of GiTim17. This technique was previously used to obtain highly specific protein profiles of the mitosomal interactome (Martincová et al. 2015). Briefly, the HSP isolated from a *Giardia* cell line expressing, in vivo, GiTim17 biotinylated by biotin ligase (BirA) (fig. 4*A*) was chemically cross-linked and GiTim17-containing complexes were purified on streptavidin magnetic beads (fig. 4*B*). The sample was analyzed by mass spectrometry and quantified against the negative control isolated from a strain expressing only BirA. For each identified protein, the enrichment ratio between the sample and the control was calculated and the proteins were ordered accordingly (supplementary table 1, Supplementary Material online). For several highly enriched proteins the enrichment ratio could not be determined, as these proteins were not identified in the control sample (fig. 4*C*). These include the bait protein Tim17, *Giardia* orthologue of Tim44 (GiTim44), two proteins of unknown function (GL50803_17276 and GL50803_10971) and *Giardia* orthologue thioredoxin reductase. The presence of Tim44, among the highly enriched proteins strongly supports the function of GiTim17 as a protein-conducting channel. In mitochondria, the protein functions as a molecular tether of the Hsp70 motor (PAM) complex to the TIM23 translocase (Kronidou et al. 1994; Ting et al. 2017). Interestingly, GiTim17 contains the conserved arginine residue responsible for Tim44 binding in yeast mitochondria (Demishtein-Zohary et al. 2017) (fig. 1*A*). However, we were not able to confirm the direct interaction between GiTim17 and GiTim44 in Y2H assays (data not shown). Whether the negative result reflects the absence of another interacting component or the experimental limitations of Y2H, requires future in vitro characterization of both proteins (Ting et al. 2017). According to the current model, the protein transport machinery across the inner mitosomal membrane involves channel-forming GiTim17, four components of the PAM motor complex: mtHsp70, its nucleotide release factor Mge1, Pam16 and Pam18 and finally Tim44, connecting the channel with the motor. The import of proteins to the mitosomes is followed by the processing of N-terminal targeting presequences by unique single subunit matrix processing peptidase (βMPP) (Šmíd et al. 2008), which was likewise also highly copurified with GiTim17. None of the other mitochondrial Tim proteins could be identified in the data set, which is supported by their absence in other metamonada representatives (Leger et al. 2017).

Analogously to the original study introducing the biotin based purification of mitosomal proteins upon chemical crosslinking (Martincová et al. 2015), the isolation of GiTim17 crosslinks served also as a general probe of the mitosomal proteome. Thus, in addition to multiple components of ISC pathway, which represent the functional core of the mitosomal metabolism, several putative new mitosomal proteins were found among the top copurified proteins (fig. 4*C*). These include above mentioned thioredoxin reductase, a potential antigiardial drug target (Leitsch et al. 2016), molecular chaperone ClpB, NEK kinase and a protein of unknown function GL50803_3098. The characterization of possible role of these components in the mitosomal protein import or other aspects of mitosome biology is a matter of exciting future studies.

**Fig. 5.**
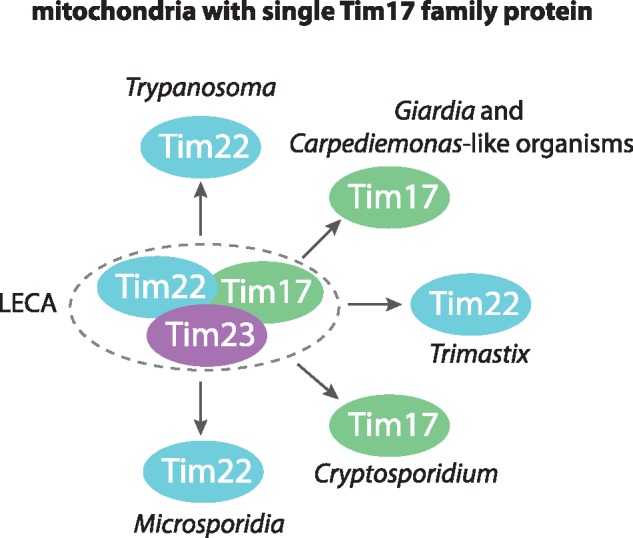
—Schematic representation of mitochondria converging on a single Tim17 family protein translocase. Distinct lineages of eukaryotes have independently reduced their mitochondrial protein import pathways to a “single Tim” translocase in the inner membrane. According to the phylogenetic reconstruction and classification of the protein family members (Žárský and Doležal 2016), these translocases were derived from either the Tim22 or Tim17 subunit.

Of the three paralogues—Tim17, Tim22, and Tim23—that mediate protein transport across the inner mitochondrial membrane, several eukaryotes have simplified the set to just a single Tim17/22/23 family protein, like *Giardia* (Žárský and Doležal 2016). Commonly, these eukaryotes have highly reduced their mitochondria to minimalist mitosomes, such as in *Giardia*-related CLOs (Metamonada) (Leger et al. 2017), Microsporidia (Burri et al. 2006), and *Cryptosporidum parvum* (Apicomplexa) (Henriquez et al. 2005). The only exception is the mitochondrion of trypanosomatids, such as *Trypanosoma brucei* (Schneider et al. 2008). Their mitochondria are complex organelles with fully developed cristae, capable of oxidative phosphorylation, and yet they contain a single Tim17/22/23 family protein. This protein has been verified as an inner membrane transporter (Singha et al. 2008) and functions in complex with several trypanosome-specific proteins (Singha et al. 2012). Similarly, *Giardia*-specific proteins of unknown function, which were copurified with GiTim17, may represent components of a lineage specific protein import apparatus. Evidently, the evolutionarily independent reduction of mitochondria also manifests as convergence on a “single Tim17 family protein translocase.” On the basis of the recent classification of the Tim17/22/23 protein family and the suggested presence of all three paralogues in the last eukaryotic common ancestor (LECA) (Žárský and Doležal 2016), it appears that the “single Tim” design is not derived from only one paralogue (fig. 5). That the “single Tim” of *Trimastix*, microsporidia, and kinetoplastids is likely derived from Tim22, whereas that of *C. parvum*, *Giardia*, and CLOs is from Tim17, indicates that both proteins have the capacity to build functional protein-conducting channels.

## Materials and Methods

### Bioinformatics

The HMM profile of Tim17 protein and the hmmer3 program (Eddy 2011) were used to repeatedly search all available genomic and transcriptomic data from diplomonads and their free-living relatives (CLOs). At every step, the HMM profile was enriched for new sequences with high scoring hits (default value by hmmer3). The third round of searches yielded the GiTim17 candidate sequence and it was also the last round of searches to yield any new sequences. Representative sequences of Tim17/22/23 family proteins across the diversity of eukaryotes (Žárský and Doležal 2016) as well as all Tim17 sequences recovered from diplomonads and CLOs were aligned using the mafft-linsi (Katoh and Standley 2013) algorithm. The resulting alignment was then manually edited and ambiguously aligned regions were manually identified and trimmed (full and trimmed alignments are available in DataDRYAD repository, https://doi.org/10.5061/dryad.1p67145). A phylogenetic tree was reconstructed using RAxML with LG+G model and statistical support was inferred from 500 bootstrap replicates. Hydrophobicity profiles and TMD predictions were inferred using TMHMM (Krogh et al. 2001) and Phobius (Käll et al. 2007). HHPRED predictions were completed using the online interface at https://toolkit.tuebingen.mpg.de/#/tools/hhpred.

### Cell Culture and Fractionation

Trophozoites of *G. intestinalis* strain WB (ATCC 30957) were grown in TY-S-33 medium (Keister 1983) supplemented with 10% heat-inactivated bovine serum (PAA Laboratories), 0.1% bovine bile, and antibiotics. Cells containing BirA were grown in medium supplemented with 50 µM biotin.

### Cloning and Transfection

Table S2, Supplementary Material online in the supplemental material lists all primers used in this study. For determination of cellular localization, the GL50803_10452 gene was amplified from genomic DNA and subcloned into a pTG vector containing an HA-tag (Martincová et al. 2012) using NdeI and PstI restriction sites. For the biotinylation assay, we used a pTG plasmid containing *E. coli* BirA and the GL50803_10452 gene was subcloned to pONDRA with a C-terminal BAP-tag using NdeI and XhoI restriction sites (Martincová et al. 2015). Transfection was performed as previously described (Voleman et al. 2017). Genes for Y2H were PCR amplified and subcloned into pGADT7 and pGBKT7 vectors using NdeI (AseI) and BamHI (BglII) restriction sites.

### Fluorescence Microscopy

*Giardia* *intestinalis* trophozoites were fixed with 1% formaldehyde as previously described (Dawson et al. 2007). The GL50803_9296 protein was recognized by a specific antibody produced in rabbit and the hemagglutinin epitope (HA tag) was recognized by a rat monoclonal antibody (Roche). Primary antibodies were detected by donkey Alexa 594 (red)-conjugated antirabbit antibodies and donkey Alexa 488 (green)-conjugated antirat antibodies (Life Technologies). Alexa 488 (green)-conjugated streptavidin (Life Technologies) was used to detect biotinylation. Slides were mounted with Vectashield containing DAPI (Vector Laboratories). Slides were imaged with an OLYMPUS Cell-R, IX81 microscope system, and the images were processed using ImageJ 1.41e software (NIH). STED microscopy was performed on a commercial Abberior STED 775 QUAD Scanning microscope (Abberior Instruments GmbH, Germany) equipped with Ti-E Nikon body, QUAD beam scanner, Easy3D STED Optics Module, and Nikon CFI Plan Apo Lambda objective (60x Oil, NA 1.40). Samples were illuminated by pulsed 561 nm and 640 nm lasers and depleted by a pulsed 775 nm STED laser of 2 D donut shape (all lasers: 40 MHz repetition rate). Fluorescence signal was detected with single photon counting modules (Excelitas Technologies). Line-interleaved acquisition enabled separated detection of individual channels in spectral range from 605 nm to 625 nm and from 650 nm to 720 nm. The confocal pinhole was set to 1 AU.

### Coimmunoprecipitation and Mass Spectrometry

*Giardia* *intestinalis* Tim17-BirA cells were grown in standard medium supplemented with 50 M biotin for 24 h prior to harvesting. Cells were harvested and fractionated as previously described (Martincová et al. 2015). The HSP (40 mg) was used for crosslinking and protein isolation. Crosslinking was performed as previously described (Martincová et al. 2015) using 10 µM DTME and 1 h incubation on ice. Proteins were eluted from the beads in SDS-sample buffer supplemented with 20 mM bitotin, for 5 min at 95 °C. Samples were analyzed by Western blotting using streptavidin conjugated Alexa Fluor 488 and were visualized using a Molecular Imager FX imager (Bio-Rad). Remaining eluate was analyzed with mass spectrometry.

Samples were dissolved in 100 mM TEAB (Triethylammonium bicarbonate, Thermofisher) buffer with 2% sodium deoxycholate (SDC, Sigma) and sonicated. Samples were reduced with 5 mM TCEP (Tris(2-carboxyethyl)phosphine hydrochloride, Sigma) for 30 min at 60 °C and alkylated with 10 mM MMTS (S-Methylmethanethiosulfonate, Sigma) for 10 min at RT. Total amount of protein was measured with a BCA kit (Sigma). Hundred micrograms of protein was digested with trypsin (trypsin:protein ratio 1:50) overnight at 37 °C. After digestion, 1% TFA (Trifluoroacetic acid, Sigma) was added. SDC was removed by extraction with ethylacetate as previously described (Masuda et al. 2008). Briefly, 200 µl of ethylacetate was added, the sample was vortexed for 1 min and centrifuged at 4,000 × g for 30 s, and the supernatant was discarded. This was repeated five times. Remaining ethylacetate was removed using vacuum centrifugation at 45 °C for 10 min. After ethylacetate removal, 1% TFA was added.

Sample desalting was performed with in house-made tip columns. Each tip was filled with three layers of C18 sorbent (Sulpeco) as previously described (Ishihama et al. 2006). Fifteen micrograms of protein was loaded on each pre-equilibrated tip. The eluted samples were dried with a vacuum dryer and resuspended in 20 µl of 1% TFA. Two micrograms of samples were used for LC/MS measurement.

A Nano Reversed phase column (EASY-Spray column, 50 cm × 75 µm ID, PepMap C18, 2 µm particles, 100 Å pore size) was used for LC/MS analysis. Mobile phase buffer A was composed of water, 2% acetonitrile, and 0.1% formic acid. Mobile phase buffer B was composed of 80% acetonitrile and 0.1% formic acid. Samples were loaded onto the trap column (Acclaim PepMap300, C18, 5 µm, 300 Å Wide Pore, 300 µm × 5 mm) at a flow rate of 15 µl/min. Loading buffer was composed of water, 2% acetonitrile, and 0.1% trifluoroacetic acid. Peptides were eluted with gradient of B from 2% to 40% over 60 min at a flow rate of 300 nl/min. Eluting peptide cations were converted to gas-phase ions by electrospray ionization and analyzed on a Thermo Orbitrap Fusion mass spectrometer (Q-OT-qIT, Thermo).

Spectra were acquired on the Orbitrap Fusion mass spectrometer (Thermo Scientific) with 2 s duty cycle. Full MS spectra were acquired in Orbitrap within mass range 350–1400 *m*/*z* with resolution 120,000 at 200 *m*/*z* and maximum injection time 50 ms. The most intense precursors were isolated by quadrupole with 1.6 *m*/*z* isolation window and fragmented using HCD with collision energy set to 30%. Fragment ions were detected in ion trap with scan range mode set to normal and scan rate set to rapid with maximum injection time 35 ms. Fragmented precursors were excluded from fragmentation for 60 s.

Raw data were processed in MaxQuant LFQ (Cox et al. 2014). LFQ quantification was used for estimation of the relative amount of each protein. Only proteins with valid values in at least two replicates (in control or treated group) were used for further processing. Searches were done in the latest version of the *G. intestinalis* database from EuPathDb (http://eupathdb.org/eupathdb/) and a common contaminant database. Modifications were set as follows: Cystein (unimod nr: 39) as static, and methionoine oxidation (unimod: 1384) and protein N terminus acetylation (unimod: 1) as variable. Further data processing of the MaxQuant results was done in Perseus (Tyanova et al. 2016).

### Y2H Assay

Yeast strain AH109 was inoculated into 5 ml 2xYPAD media and incubated over night at 30 °C, 200 RPM. Grown culture was diluted with 2xYPAD medium to OD_600_ = 0.2 and incubated (30 °C, 200 RPM) until OD_600_ = 0.8. Cells were harvested (3,000 × g, 5 min), washed in H_2_O, and resuspended in 1 ml H_2_O. The carrier DNA (Salmon sperm) was denatured (95 °C, 5 min) and placed on ice. Cells were pelleted (3,000 × g, 1 min) and the supernatant was discarded. Hundred microliters of the cells were resuspended in 400 ml H_2_O and divided into separate tubes—50 µl for each transformation. Cells were pelleted (3,000 × g, 1 min) and the supernatant discarded. Solutions were added to tubes in the following order: 240 µl PEG3500 50% w/v, 36 µl 1 M LiAc, 50 µl Salmon sperm DNA, 5 + 5 µl of each plasmid DNA, 24 µl H_2_O. Each transformation was vortexed thoroughly for 1 min. Transformed cells were incubated in 42 °C, 40 min, then pelleted (3,000 × g, 1 min) and the supernatant discarded. Yeast cells were resuspended in 500 µl H_2_O, divided into two, and spread on SD-Trp/-Leu plates with kanamycin (50 µg/ml; for verification of successful transformation) and on SD-Trp/-Leu/-His plates with kanamycin (for interaction test). Plates were incubated in 30 °C until colonies appeared (3–4 days).

### Serial Dilution Test

One colony was inoculated into 5 ml SD-Trp/-Leu with kanamycin (50 µg/ml) and incubated overnight at 30 °C, 200 RPM. Cells were pelleted (3,000 × g, 1 min) and the supernatant discarded. Yeast were resuspended in H_2_O to OD_600_ = 0.2 and a serial dilution was made (40 µl of cell suspension in 160 µl H_2_O, 20×–200,000×). Two microliters of each dilution was dropped on SD-Trp/-Leu, SD-Trp/-Leu/-His, and SD-Trp/-Leu/-His with 30 mM 3-Amino-1, 2, 4-triazole (3-AT) plates, which were incubated at 30 °C until colonies appeared.

### In Vitro Protein Expression

GiTim17 was synthesized in vitro using the PURExpress In Vitro Protein Synthesis Kit (NEB). The gene was cloned to the DHFR control plasmid (provided in the Kit). The 25 µl translation reaction contained: 10 µl solution A; 7.5 µl solution B; 250 ng template DNA; 1 µl RNase inhibitor (RNAsin, Promega); radioactively labelled ^35^S-methionin; and 50 µg lecithin liposomes. Liposomes were prepared from stock solution of soybean 1-α-lecithin in chloroform by evaporating the chloroform under a nitrogen flow, resuspending the lipid film in dH_2_O, and subsequent sonication in a water bath sonicator. The translation reaction was incubated for 2 h at 37 °C and then centrifuged for 45 min at 13,000 × g. The pellet was resuspended in 50 mM sodium phosphate buffer (pH = 8) with 2 M urea, centrifuged, and then washed in clear 50 mM sodium phosphate buffer. The output was analyzed on Blue Native PAGE, using 2% digitonin and NativePAGE Novex 4-16% Bis-Tris Protein Gel (Thermo Fisher Scientific).

## Supplementary Material

Supplementary data are available at *Genome Biology and Evolution* online.

## Supplementary Material

Supplementary Data
