# Supplementary material for: A Single Tim Translocase in the Mitosomes of Giardia intestinalis Illustrates Convergence of Protein Import Machines in Anaerobic Eukaryotes
Source: Genome Biol Evol. 2018 Sep 28;10(10):2813–22. doi: 10.1093/gbe/evy215 (PMC6200312; doi:10.1093/gbe/evy215)
Supplement: Supplementary Data [file evy215_supp.zip › Supplementary figure legends.docx]

**Supplementary Data**

**Supplementary Figure 1 Phylogenetic tree of Tim17 family proteins**

**Supplementary Figure 2 GRAVY analysis of Tim17 family proteins**

886 sequences of the Tim17 protein family were analysed for their grand average of hydropathy (GRAVY) (http://www.gravy-calculator.de); results are displayed in a box-and-whisker plot. Red line depicts the value of GiTim17.

**Supplementary Table 1 Mass Spectrometry analysis of Tim17-BAP co-precipitation.**

**Supplementary Table 2 Primers used in the study**
