# Supplementary material for: A Single Tim Translocase in the Mitosomes of Giardia intestinalis Illustrates Convergence of Protein Import Machines in Anaerobic Eukaryotes
Source: Genome Biol Evol. 2018 Sep 28;10(10):2813–22. doi: 10.1093/gbe/evy215 (PMC6200312; doi:10.1093/gbe/evy215)
Supplement: Supplementary Data [file evy215_supp.zip › Supplementary table 2.docx]

Table S1 Primers used in the study

| primer | sequence | vector |
| --- | --- | --- |
| GL50803_10452 NdeIF | CATGCATATGTTAAGGCTCTGGCAA | pTG |
| GL50803_10452 PstIR | CTAGCTGCAGCGCCTTCTTGTCCATCTC | pTG |
| GL50803_10452 NdeIF | CATGCATATGTTAAGGCTCTGGCAA | pONDRA |
| GL50803_10452 BAP XhoIR | CTAGCTCGAGTAATTCGTGCCATTCTATGGCCTGTGCCTCAAAGATATCATTTAAGCC | pONDRA |
| GL50803_10452 NdeIF | CTAGCATATGTTAAGGCTCTGGCAA | pGADT7/pGBKT7 |
| GL50803_10452 BamHIR | CATGGGATCCCTACGCCTTCTTGTCCAT | pGADT7/pGBKT7 |
| GL50803_10452 NdeIF | CTAGCATATGTTAAGGCTCTGGCAA | pDHFR |
| GL50803_10452 BamHIR | CATGGGATCCCTACGCCTTCTTGTCCAT | pDHFR |
