# Supplementary figures and images for: A Single Tim Translocase in the Mitosomes of Giardia intestinalis Illustrates Convergence of Protein Import Machines in Anaerobic Eukaryotes
Source: Genome Biol Evol. 2018 Sep 28;10(10):2813–22. doi: 10.1093/gbe/evy215 (PMC6200312; doi:10.1093/gbe/evy215)

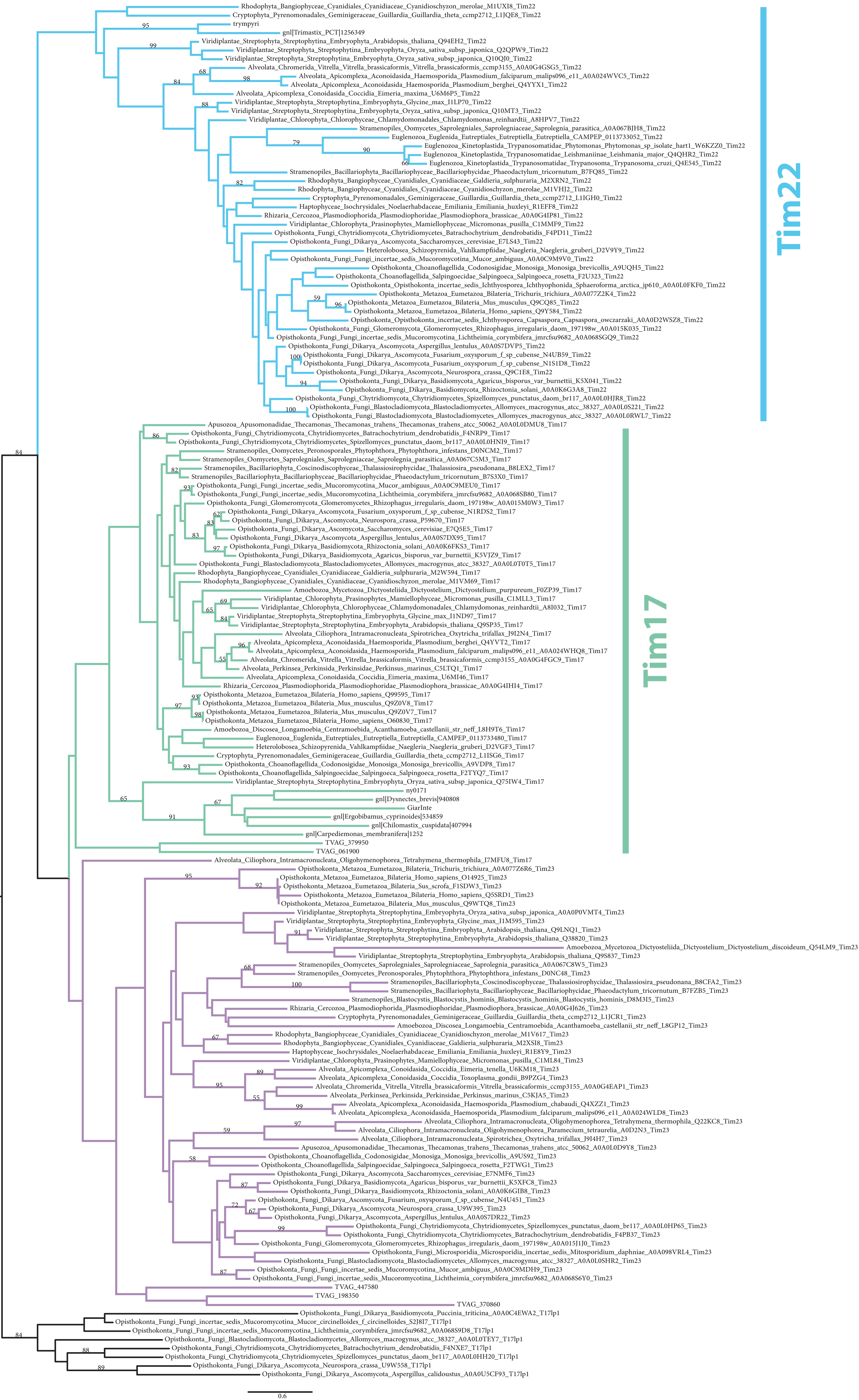

Tim22

Tim17

Tim23

Supplement: Supplementary Data [file evy215_supp.zip › Supplementary Figure 1.pdf]

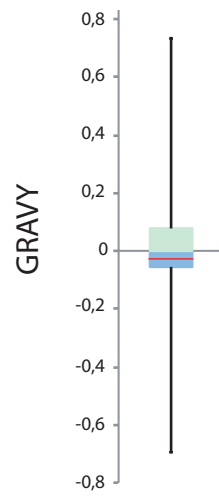

Supplementary Figure 2

Supplement: Supplementary Data [file evy215_supp.zip › Supplementary Figure 2.pdf]
